# Assessing animal welfare in invasive bird management: evidence for reduced stress hormones with lethal shooting

**DOI:** 10.1093/conphys/coag025

**Published:** 2026-04-22

**Authors:** Isabel López-Rull, Jon Blanco-González, Diego Gil, Alejandro Calvo Gómez, Fernando Enríquez, Luis Cayuela

**Affiliations:** Área de Biodiversidad y Conservación, Universidad Rey Juan Carlos, C/Tulipán s/n., E-28933 Móstoles, Spain; Instituto de Investigación en Cambio Global, Universidad Rey Juan Carlos, C/Tulipán s/n., E-28933 Móstoles, Spain; Área de Biodiversidad y Conservación, Universidad Rey Juan Carlos, C/Tulipán s/n., E-28933 Móstoles, Spain; Departamento de Ecología Evolutiva, Museo Nacional de Ciencias Naturales (CSIC), C/ José Gutiérrez Abascal 2, Chamartín, 28006, Madrid, Spain; Área de Biodiversidad y Conservación, Universidad Rey Juan Carlos, C/Tulipán s/n., E-28933 Móstoles, Spain; Departamento Forestal, Mantenimiento de infraestructuras S.A.U. MATINSA. C/ de Federico Salmón, 13, Chamartín, E-28016 Madrid, Spain; Área de Biodiversidad y Conservación, Universidad Rey Juan Carlos, C/Tulipán s/n., E-28933 Móstoles, Spain; Instituto de Investigación en Cambio Global, Universidad Rey Juan Carlos, C/Tulipán s/n., E-28933 Móstoles, Spain

**Keywords:** Animal confinement, animal transportation, control methods, corticosterone, glucocorticoids, live trapping, monk parakeet, shooting, stress response

## Abstract

Refinement of management actions for invasive species requires identifying methods that minimize physiological strain on target animals. Glucocorticoid concentrations are commonly used to assess the activation of the hypothalamic–pituitary–adrenal (HPA) axis in response to acute stress. Using monk parakeets (*Myiopsitta monachus*) as an avian invader model, we aimed to assess the HPA response of two common control methods: shooting and live trapping followed by confinement until euthanasia. Plasma corticosterone (CORT), the primary avian glucocorticoid, was measured in 63 parakeets assigned to one of three groups: (i) shooting (*N* = 7), (ii) baseline (live-trapped birds in which blood samples were taken <3 minutes after capture, *N* = 6) and (iii) live trapping–confinement (live-trapped birds in which blood samples were collected 10–210 minutes after capture, *N* = 50). Differences among treatments were analysed using a generalized linear model with a gamma error distribution, and CORT dynamics over time were evaluated with non-linear logistic and quadratic models. CORT levels in shot birds were similar to baseline, indicating that their death occurred before a systemic HPA axis response was initiated. In contrast, live-trapped–confined birds showed markedly elevated CORT levels. The logistic model best described the stress response, showing CORT rising with time after capture until stabilizing while the stressor persisted. These results have important implications for management design. Since shooting has been proved effective in population reduction and we found it is associated with reduced CORT levels compared to trapping, we recommend that practitioners prioritize this method when controlling monk parakeets. By incorporating these objective metrics, managers can offer transparent, evidence-based justifications for the selection of control techniques, potentially reducing social conflict and fostering greater public acceptance of managing protocols for invasive species. Efforts should be made to inform the public about how different bird control techniques vary in terms of the physiological impact they may cause.

## Introduction

Invasive alien species are among the main drivers of biodiversity loss and ecosystem function alteration (Sala *et al.*, 2000; Pejchar and Mooney, 2009). While mitigating their negative impacts is essential, practitioners often face public opposition to wildlife control due to evolving societal attitudes towards animals and concerns that common control actions are inhumane, ineffective or not based on scientific evidence (Dubois *et al.*, 2017). Therefore, implementation of control actions must prioritize animal welfare, not only to improve public acceptance but also to uphold ethical standards.

Welfare describes an individual’s psychological experience, shaped by continuous interactions with both internal and external factors (Broom and Johnson, 1993; Fraser, 1999; Rushen, 2003; Wu *et al.*, 2025). While defining a state of optimal welfare remains a complex challenge, identifying the factors that compromise it is more attainable. By addressing the prerequisites necessary to prevent negative physical and psychological effects, researchers can establish ethical standards to maintain welfare to the best of our current knowledge (Wielebnowski, 2003; Wu *et al.*, 2025 for a historical perspective). When implementing invasive species control programmes, these standards should be upheld to the greatest extent possible, ensuring that even when lethal control actions are necessary, they are conducted with the highest possible regard for the animal’s well-being (Broom *et al.*, 1999).

Because animal emotions cannot be measured directly, indices that typically reflect internal states, such as behavioural and physiological responses to stress, are commonly used (Mormède *et al.*, 2007; National Research Council [US] Committee on Recognition and Alleviation of Distress in Laboratory Animals, 2008). Physiological stress can be defined as the multidimensional physiological response to predictable and unpredictable environmental stimuli (stressors) that challenge internal homoeostasis (Dantzer *et al.*, 2014). The two primary physiological responses to stressors are the stimulation of the sympathetic nervous system, resulting in the release of catecholamines, and the activation of the hypothalamic–pituitary–adrenal (HPA) axis (Sapolsky *et al.*, 2000; Reeder and Kramer, 2005). The latter regulates the synthesis and secretion of glucocorticoid hormones with effects lasting from several minutes to hours (Wingfield and Romero, 2010). At baseline levels, glucocorticoids primarily regulate metabolic functions, such as energy intake. However, under acute stress, glucocorticoids rapidly rise, causing behavioural modifications and energy mobilization as the individual attempts to cope with adverse conditions (Sapolsky *et al.*, 2000). The cessation of the pathway is under the control of the glucocorticoids themselves through a negative feedback loop: when the stressor ends, the system facilitates a rapid return to homoeostasis, restoring hormonal levels to their baseline state (Sapolsky *et al.*, 2000).

Control methods employed in invasive species management are documented to induce varying degrees of fear, pain or social disruption (Broom *et al.*, 1999; Dubois *et al.*, 2017). Given these potential impacts, measuring circulating glucocorticoids serves as a primary indicator of the physiological strain experienced by target animals. While measuring these hormones is the standard approach to the study of stress and welfare in animals (Mormède *et al.*, 2007), it is important to acknowledge that the relationship between glucocorticoids and animal welfare is complex, since elevated levels reflect physiological arousal that may not always signify a compromised welfare state (Mormède *et al.*, 2007; Ralph and Tilbrook, 2016). Ideally, a comprehensive assessment of welfare would integrate glucocorticoid levels with complementary metrics, such as behavioural changes or physical health indicators (Ralph and Tilbrook, 2016). In the context of invasive species management, behavioural observations are often precluded by logistical constraints, particularly during large-scale captures or *in situ* lethal control of free-living animals, where interventions are prioritized for speed and efficiency. Under these conditions, the elevation in glucocorticoid levels, although not a perfect proxy, may provide an objective physiological measure of the acute stress response induced by specific control methods. In fact, glucocorticoids remain the most widely used biomarkers for quantifying the magnitude of the HPA response to acute stressors (Mormède *et al.*, 2007; Dickens and Romero, 2013; Alquezar *et al.*, 2023; Wu *et al.*, 2025). The diagnostic value of these measurements, however, depends on the timing of blood collection relative to the stressor’s onset. Glucocorticoid levels typically increase in the blood within 3–5 minutes of the onset of a stressful event, reaching the peak within 15–30 minutes and returning to baseline activity about 60–90 minutes after the stressor ends (Sheriff *et al.*, 2011). To ensure accurate interpretation, baseline levels must be assessed within 3 minutes of the initial disturbance to avoid the confounding effects of the handling-induced rise (Romero and Romero, 2002), whereas the magnitude of the acute stress response should be assessed by measuring glucocorticoid levels at least 10 minutes after exposure.

Within the context of global biological invasions, avian species represent a significant proportion, with ~400 species introduced worldwide (Kumschick *et al.*, 2016). Among them, Psittaciformes (parrots) are one of the most impactful in Europe, both ecologically and economically (Kumschick and Nentwig, 2010; Kumschick *et al.*, 2016). Impacts include disease transmission and competition with native fauna, documented in 72% of invasive psittacine species (Kumschick *et al.*, 2016). Consequently, local authorities implement control measures, including lethal methods, though these often generate social conflicts (Larraechea *et al.*, 2025), complicating management efforts. Currently, two effective control methods commonly used for invasive birds include: (i) shooting with pre-charged air rifles and (ii) live trapping followed by confinement and transportation until euthanasia (Klug *et al.*, 2023). Lethal shooting, however, is socially controversial due to concerns about public safety, acceptance in urban areas (Klug *et al.*, 2023) and perceived animal suffering. This implies that live trapping and posterior euthanasia is often prioritized as the less cruel and more humane method in current protocols. Using the monk parakeet (*Myiopsitta monachus*) as a model avian invader, this study aims to identify which of these methods is best in terms of animal welfare, assessed by glucocorticoid rise. To achieve this, we measured circulating plasma levels of corticosterone (the avian glucocorticoid, hereafter CORT) induced by each method. Baseline CORT levels were also estimated in a group of parakeets sampled by live trapping (but not confined) and bleed within 3 minutes of capture. Because shooting is intended to cause immediate death, we predicted that CORT levels in shot parakeets would be similar to baseline, whereas live-trapped–confined individuals would show elevated CORT concentrations increasing with time since capture.

## Materials and methods

### Study species and study site

The monk parakeet (*M. monachus*) is a medium-sized, sexually monomorphic parrot native to Paraguay, Uruguay, Bolivia, southern Brazil and Argentina. Driven by the pet trade, the species has been introduced worldwide and has established populations in 26 countries (Preston *et al.*, 2021) with numbers currently increasing globally (Royle and Donner, 2021). In Europe, Mediterranean countries are experiencing higher exponential growth, spread rate and faster colonization of new areas than Atlantic countries (Postigo *et al.*, 2019), with Spain hosting the largest populations, particularly in the regions of Madrid, Catalonia and Andalusia (Carrete *et al.*, 2021). Although most parakeets inhabit urban areas, they frequently travel to rural areas to feed (Senar *et al.*, 2016; Castro *et al.*, 2022), and they have begun colonizing these areas (Hernández-Brito *et al.*, 2020), thereby increasing their potential to invade and impact natural ecosystems. Due to the threats that this species poses to native biodiversity (Menchetti and Mori, 2014) and the economic impacts it causes (Senar *et al.*, 2016; Castro *et al.*, 2022), it is listed in the Spanish Catalogue of Invasive Alien Species (Royal Decree 630/2013), which requires competent authorities to implement management, control and eradication measures.

The study was conducted during November and December 2024 across various parks and green spaces in the southern part of Madrid city, Spain. The city covers an area of 604 km^2^ at an average elevation of 657 m, characterized by a continental Mediterranean climate. Madrid hosts one of Europe’s largest monk parakeet populations, with an estimated 11 154–12 975 individuals recorded in the 2019 census (Nebreda *et al.*, 2019). A management programme based on culling adults and nests was implemented from May 2021 to April 2023, reducing the projected 2023 population by 50% (Blanco-González *et al.*, 2025). However, the population is expected to recover to pre-control levels by 2025, with an estimated 18 000 reproductive individuals (Blanco-González *et al.*, 2025).

### Field procedures and sample collection

Circulating levels of CORT were quantified in 63 parakeets assigned to one of the three groups: (i) shooting (*N* = 7), (ii) baseline (live-trapped birds in which blood samples were taken <3 minutes after capture, *N* = 6) and (iii) live trapping–confinement (live-trapped–confined birds in which blood samples were collected 10–210 minutes after capture, *N* = 50).

To evaluate the CORT response in the shooting treatment, parakeets were culled using a pre-charged pneumatic (PCP) air rifle equipped with a telescopic sight and loaded with a single round-nosed pellet. This method was carried out exclusively in secure and controlled areas by a trained shooter. Immediately after culling, a blood sample was collected from the wound site. Culled parrots were then transported to a veterinary centre authorized to incinerate corpses in accordance with current legislation.

Live trapping consisted of capturing parakeets during ground-feeding using both folding nets and handheld net launchers. Folding nets (4×6 m) were discreetly installed at ground level in a fixed location throughout the day and remotely triggered to close. Parakeets were attracted to folding nets using bait consisting of bread and commercial parrot feed. Handheld net launchers are portable devices that deploy a net (1×1 m) propelled by CO_2_, allowing the capture of parakeets in various locations while foraging on the ground. The time of net activation (either folding nets or hand-held net launchers) was recorded as the onset of the stressful event. Immediately after capture, parakeets were carefully removed from the net and placed into individual cotton bags until blood sampling. Depending on the time the blood sample was taken, live-trapped parakeets were assigned to either the baseline treatment or the live trapping–confinement treatment. Parakeets assigned to the baseline treatment were blood–sampled within 3 minutes of net activation; therefore, they are assumed not to have developed a glucocorticoid-mediated stress response. In contrast, parakeets in the live trapping–confinement treatment were sampled at different time intervals, simulating live trapping management protocols. Typically, these protocols imply holding captured animals and transporting them to a designated centre for euthanasia. This holding/transportation period can last several hours (personal observation). To accurately simulate these conditions and obtain CORT measures across a range of post-capture intervals, parakeets in the live trapping–confinement treatment were held in individual bags for 10 to 210 minutes after capture. Each parakeet was sampled once within this time interval. The exact time of blood collection was recorded for each individual, ensuring a balanced distribution of individuals across the sampling range. Blood samples (1 ml) for all live-trapped parakeets were collected using a heparinized syringe. After blood collection, parakeets were released at their respective capture sites.

Blood from all sampled individuals was stored at 4°C for the rest of the morning. Upon transport to the laboratory, samples were centrifuged at 2500 rpm, and the resulting plasma aliquots and red blood cell fraction were frozen at −80°C until hormonal analyses and sex determination were conducted. To assess whether the CORT response varied by sex, sex was determined from blood samples using molecular methods (females *N* = 30; males *N* = 34).

### Hormone analysis and sex determination

CORT concentrations were determined using a highly specific enzyme immunoassay (EIA) kit (DRG Labs). Assays were performed in accordance with the manufacturer’s instructions without extraction. As the first step, we performed an in-house analytical validation by linearly diluting monk parakeet plasma. The results showed linearity and parallelism with the kit standard curve, indicating no significant interference from matrix effects (Fig. S1). Samples were diluted 1:4 with DRG steroid-free serum (STD0-DRG) and analysed in duplicate against a standard curve. The intra-assay coefficient of variation (CV) was 12.05%, and the inter-assay CV was 10.11%. Golden sample aliquots were added to the plates to calculate the inter-assay CV. DNA for sex determination was extracted from blood using the PUREGENE protocol (Gentra Systems). Approximately, 20 ng of each DNA solution was used in a polymerase chain reaction to amplify a portion of the CHD-W gene (present only in females) and the CHD-Z gene (present in both sexes), following Griffiths *et al.* (1998).

### Data analysis

To compare differences in plasma CORT levels among treatments, we fitted a generalized linear model with a gamma error distribution, including CORT concentration as the dependent variable and treatment (shooting, baseline, live trapping–confinement) as a categorical predictor. Differences among treatments were assessed using a likelihood ratio test (LRT) comparing the full model to a null (intercept-only) model. Pairwise comparisons were conducted with *post hoc* Bonferroni tests using the *emmeans* R package (Lenth, 2024).

To analyse how CORT levels in monk parakeets change over time following capture, we fitted and compared two non-linear models with a gamma error distribution: a logistic growth model and a quadratic model. In these analyses, treatments were not differentiated, and time after capture was the sole predictor. The logistic model followed the form:


$$ \mathrm{Corticosterone}=\frac{a}{1+\exp \left(-b\ast \left(\mathrm{Time}-x\right)\right)} $$

where *a* represents the asymptotic maximum CORT concentration, *x*_0_ is the inflection point (i.e. the time at which CORT reaches half of its maximum and the rate of increase is greatest) and *b* is a slope parameter determining how rapidly levels rise around the inflection point. This sigmoidal function reflects a biologically plausible hormonal surge that stabilizes over time.

The quadratic model followed the form:


$$ \mathrm{Corticosterone}=a+b\ast \mathrm{Time}+c\ast{\mathrm{Time}}^2 $$

where *a* is the intercept (baseline CORT at time zero), *b* captures the linear effect of time and *c* reflects the curvature of the response. A negative *c* indicates a peak followed by a decline, consistent with a transient stress response.

To determine whether CORT levels changed over time, we also included a null model assuming constant levels throughout. Model comparisons were performed using the Akaike Information Criterion corrected for small sample size (AICc) to evaluate relative model support (Burnham and Anderson, 2002). When the difference in AICc was <2, the simplest model was selected. Finally, we computed pseudo-*R*^2^ values to assess the explanatory power of each model. Model assumptions were assessed by visually inspecting residuals.

Once the best-fitting model was identified, we assessed whether males and females differed in their CORT response over time following capture. To address this, we compared AICc values among models that allowed specific parameters to vary by sex against a null model that assumed both sexes exhibited the same response pattern. In addition to the null model, we fitted seven alternative models: three models where each parameter (‘*a*’, ‘*b*’ and ‘*x*_0_’) varied individually by sex; three models where pairs of parameters varied by sex (i.e. ‘*a*’ and ‘*b*’; ‘*a*’ and ‘*x*_0_’; and ‘*b*’ and ‘*x*_0_’); and one model where all three parameters varied by sex. This approach enabled us to evaluate whether sex-specific differences were best explained by variation in a single parameter, combinations of parameters or across all parameters simultaneously. In all cases, logistic models were fitted using the ‘mle2’ function from the *bbmle* package (Bolker and R Development Core Team, 2023), while quadratic models were fitted using the ‘glm’ function. All analyses were performed in R software (R Core Team, 2024).

### Ethical statement

All methods of this research were carried out in accordance with the ethical guidelines proposed for the Spanish Royal Decree 53/2013, which establishes the rules applicable for the protection of animals used in experiments and scientific research. Experimental protocols were approved by the Research Ethics Committee of the Rey Juan Carlos University (Ref:0603202312723), as the organism authorized by the Comunidad Autónoma de Madrid for evaluation of projects based on what is stated in RD 53/2013. Permissions to conduct the study were granted by the Comunidad Autónoma de Madrid (Ref: 10/805562.9/24).

## Results

Important differences in plasma CORT concentrations were observed among methods (*χ*^2^₂ = 40.24; *P* < 0.001; Table S1). Live-trapped–confined parakeets showed markedly elevated CORT levels (41.00 ± 17.20 ng/ml) compared to both baseline (3.99 ± 0.87 ng/ml; Bonferroni-adjusted *P* < 0.0001) and shot individuals (2.38 ± 0.58 ng/ml; Bonferroni adjusted *P* < 0.0001; Table S1). Shot parakeets exhibited CORT levels similar to, and potentially lower than, baseline concentrations (Bonferroni-adjusted *P* = 0.07; Table S1).

**Figure 1:**
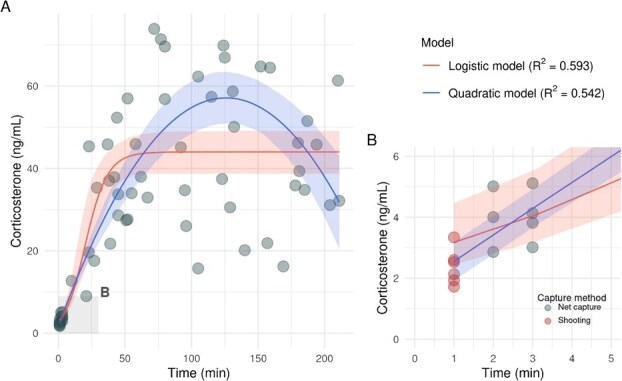
(**a**) Predicted CORT levels in monk parakeets (*M. monachus*) over time following capture, based on two non-linear models: a logistic model (red line) and a quadratic model (blue line). Both models show a rapid initial increase, followed by divergent long-term trajectories. (**b**) Zoomed view of model predictions during the first 5 minutes post-capture, highlighting the sharp early rise in CORT levels. Shaded areas represent 95% confidence intervals; points indicate observed values. The estimated model coefficients are provided in Table S2

In addition, both the logistic and quadratic models fitted the observed data well, with statistically significant improvements over the null model and pseudo-*R*^2^ values ranging from 0.542 (quadratic) to 0.593 (logistic) (Tables 1and S2). However, the logistic model had substantially stronger support, with an AICc value almost three units lower than that of the quadratic model, indicating a better fit. Despite these differences, both models exhibited a similar pattern in the first 25–30 minutes following capture (Fig. 1a), with CORT levels rising sharply—from ~5 ng/ml (Fig. 1b) at 5 minutes to ~20 ng/ml at 25 minutes (Fig. 1a). Beyond this point, the models diverged: the logistic model predicted a sharp increase in CORT, reaching ~ 45 ng/ml at 50 minutes post-capture, while the quadratic model predicted a peak of ~57 ng/ml at 2 hours, followed by a rapid decline. In both cases, variability in the data was high, though most observations showed elevated CORT levels (>20 ng/ml) after the first 25 minutes (Fig. 1a).

**Table 1:** Model comparison results evaluating the effect of time since capture on CORT levels in monk parakeets (*M. monachus*)

**Model**	**Res. Df**	**AICc**	**ΔAICc**	**Df**	**Weight**	**Pseudo-*R*** ^**2**^
Null model	62	568.0	111.4	2	<0.001	
**Logistic model**	**60**	**456.7**	**0.0**	**4**	**0.810**	**0.593**
Quadratic model	60	459.5	2.8	4	0.190	0.542

Three models were compared: a null model assuming constant CORT levels, a logistic model representing a sigmoidal increase over time and a quadratic model allowing for a rise-and-fall response. Comparisons were based on the AICc. Abbreviations: *D*f, number of estimated parameters; Pseudo-*R*^2^, proportion of variance explained by each model; Res. Df, residual degrees of freedom; Weight, relative likelihood of each model being the best among the candidate set, given the data; ΔAICc, differences in AICc relative to the best-fitting model (i.e. the model with the lowest AICc). The best model is shown in bold.

In addition to the null model, seven logistic models were fitted and compared to evaluate the effect of sex on the CORT response over time since capture (Table S3). None of the models outperformed the null model, which assumed no sex differences in the CORT response. Models allowing parameters ‘*x*_0_’, ‘*b*’ or the combination of ‘*x*_0_’ and ‘*a*’ to vary between sexes showed similar AICc values, while the remaining models had higher AICc (Table S3).

## Discussion

Here, we aimed to assess the stress response mediated by glucocorticoids in monk parakeets exposed to two different methods of bird control. We observed great differences between methods, with shooting leading to lower mean plasma CORT levels than live trapping and posterior confinement. Notably, we found that shot parakeets exhibited CORT levels that were statistically indistinguishable from, and potentially lower than, baseline concentrations. While the small sample size in these groups may have limited the statistical power to detect a significant difference, the fact that levels remained at or below baseline values is biologically relevant. This suggests that the lethal shooting method is sufficiently rapid to prevent the initiation of the HPA response, potentially even avoiding the minor physiological arousal typically associated with the capture and handling required for baseline blood sampling. In contrast, live-trapped parakeets showed elevated CORT levels, which varied as a function of the time elapsed from capture to blood sampling. These results have important implications for the design and implementation of parrot control programmes, since our data indicate that shooting minimizes physiological strain as measured by plasma CORT levels compared to live trapping–confinement.

Our results showing how the CORT levels in monk parakeets change over time following live trapping suggest that this method has consequences for animal welfare. Interpreting an animal’s response to stress requires knowledge of baseline concentrations of CORT, which reflect pre-stress hormone levels (Wingfield and Romero, 2010). For a variety of avian species, samples taken less than 2–3 minutes after capture are known to provide reliable baseline, unstressed CORT concentrations (Schoech *et al.*, 1999). In our study population, baseline concentrations of CORT were less than ~5 ng/ml, similar to those reported for other avian species, including psittacids (Parks *et al.*, 2023; Quirici *et al.*, 2023). After the first 25–30 minutes following live trapping, CORT levels increased to ~20 ng/ml and then, depending on the model, CORT reached and remained at ~ 45 ng/ml at 50 minutes post-capture (logistic) or reached a peak of ~57 ng/ml at 2 hours, followed by a rapid decline (quadratic). While both models fit the data distribution, the stress response of the individuals in our study is best explained by the logistic model, which reflects that the response persists while the stressor is present. This persistent elevation suggests that, although the negative feedback loop of the HPA axis is designed to inhibit further secretion of CORT and restore homoeostasis (Sapolsky *et al.*, 2000), the continuous nature of the stressor, in this case confinement, likely prevents the system from returning to baseline levels. In such a scenario, the HPA axis may reach a state where the drive of the stressor overcomes the inhibitory feedback signal. Although monk parakeets in our study were not euthanized, we subjected them to a period of captivity similar to the holding time birds experience before euthanasia in control operations. Throughout this interval, the parakeets continued to exhibit elevated CORT levels. During trapping, parakeets can suffer injuries while trying to escape or when being removed from nets. Furthermore, it is expected that the time elapsed between capture and euthanasia—often involving hours of confinement in carriers (which are frequently not individualized, leading to crushing, pecking and increased temperature)—causes high levels of stress to the birds. The deprivation of food and water during this time, combined with the perception of stress vocalizations from their conspecifics, as these are highly social animals that communicate continuously, results in negative affective states like hunger, thirst and nervousness. It is important to acknowledge that subsequent stages of management, such as transport to holding facilities, may impose additional stress. Previous research indicates that transport can have significant physiological consequences for birds (reviewed in Fazio and Ferlazzo, 2003; Dickens *et al.*, 2010), potentially exacerbating the adrenocortical response beyond that observed during simple confinement in holding bags. Future research should prioritize sampling individuals throughout the transport process to determine if these effects further widen the gap between live-capture methods and lethal shooting. Furthermore, while our glucocorticoid data provide a significant measure of acute stress, future studies should integrate endocrine data with a broader suite of indicators such as fine-scale behavioural observations, heart rate variability or oxidative stress to build a more comprehensive understanding of the welfare implications of parakeet control.

We found that the glucocorticoid patterns were similar between sexes. In birds, sex differences in CORT levels are often linked to specific sex roles during breeding, with lower CORT levels typically observed in the sex that performs more parental care (O'Reilly and Wingfield, 2001). In monk parakeets, the male performs most of the nest construction and feeds the female during the incubation and early nestling period, while incubation is performed mostly by the female and both sexes feed the nestlings (Eberhard, 1998). Given that all individuals in our study were sampled outside of the breeding season and that both sexes contribute significantly to raising offspring, we did not expect to find sex-specific differences in their CORT levels.

Our results show that shooting caused no increase in CORT levels. Shooting is a widely employed tool in wildlife control (Caudell *et al.*, 2009; Bengsen *et al.*, 2020) and is recognized in international management protocols as a rapid and effective method when performed by trained professionals (PestSmart., 2020). It has proven successful in most parakeet management programmes (Spain: Orueta, 2007 (Balearic Islands), Molina *et al.*, 2016 (Balearic Islands), Esteban, 2016 (Zaragoza), Blanco-González *et al.*, 2025 (Madrid); Uruguay: Bruggers *et al.*, 1998, Linz *et al.*, 2015; USA**:**  Neidermyer and Hickey, 1977, Avery and Shiels, 2017). Additionally, recent decision-making models show that shooting is the most effective and cost-efficient method to cull monk parakeet populations (Senar *et al.*, 2021; Blanco-González *et al.*, 2025). Indeed, shooting can be particularly useful for (i) targeting sexually mature adults, which are main objective for population control given their higher survival rate into subsequent breeding seasons (Conroy and Senar, 2009), (ii) managing populations where individuals have developed net-avoidance behaviours (Klug *et al.*, 2023) and (iii) eliminating incipient monk parakeet populations, thereby preventing their dispersal and proliferation. Some examples of the latter include the Spanish locations of Zaragoza, in which shooting resulted in the successful eradication of the parakeet population (Esteban, 2016), and the Balearic Archipelago, in which a combination of control methods, including shooting, achieved near-total eradication of monk parakeets (Postigo *et al.*, 2019). Furthermore, a study on monk parakeet distribution and population sizes in the European Union found that, among 179 municipalities in which parakeets are present, 84% had small populations (Postigo *et al.*, 2019), for which shooting would be a highly effective control method given the advantages outlined above. We acknowledge that shooting individuals, rather than capturing entire social units at once via netting, could potentially disrupt social bonds or increase anxiety in a manner similar to overhunted species. However, while future studies are needed to clarify these long-term social impacts, the data presented here indicate that shooting can be considered an ethically justifiable method due to its ability to prevent the severe and prolonged physiological strain associated with capture and confinement. Despite these advantages over other lethal methods, in urban areas shooting often faces public opposition. Control initiatives targeting charismatic invasive species, particularly those introduced through the pet trade, frequently lead to conflict regarding management strategies due to the affinity towards species perceived as companion animals (Bidegain *et al.*, 2024). Specifically, concerning monk parakeets, a recent study evaluating public attitudes towards control measures (Larraechea *et al.*, 2025) found that while over 70% of the 504 respondents supported a control programme, nearly 59% found culling unacceptable. Furthermore, 78.2% indicated that their decision to support such initiatives would be influenced by the chosen methods, with shooting (71%) and live trapping followed by euthanasia (63%) being highly rejected options. These public preferences are consistent with previous research that reflects ethical concerns related to perceived cruelty, selectivity and unintended risks (Bremner and Park, 2007).

In this context, it is important to acknowledge that improper shooting can lead to animal welfare issues, particularly when individuals are wounded but not killed outright, causing them to suffer pain and stress before potentially dying or recovering (Baker and Harris, 2005). However, the use of glucocorticoid levels as a direct proxy for ‘suffering’ in these scenarios requires careful nuance. While our results show that lethal shooting maintains CORT at levels comparable to baseline, these findings primarily reflect the immediacy of the method, causing death before the HPA axis can mount a systemic response. In this regard, it is essential to distinguish between the immediate sympathetic nervous system response and the subsequent activation of the HPA axis. The release of catecholamines and the perception of pain occur almost instantaneously upon injury or perceived threat. In contrast, circulating CORT levels typically require several minutes to show a significant increase. Therefore, the baseline-like levels observed in shot individuals in this study do not imply the absence of the expected immediate nervous system discharge or nociceptive signalling; rather, they demonstrate that death occurred before the HPA axis could mount a systemic response. While we acknowledge that CORT data represent only one facet of the physiological response, maintaining HPA cascade activity at baseline levels remains a valuable indicator of a reduced period of physiological strain during the procedure. It is critical to recognize that a baseline-like CORT measurement does not prove the absence of suffering; rather, it indicates a time course shorter than the 2–3-minute window typically required for circulating glucocorticoids to rise. Consequently, the welfare benefits of lethal shooting are intrinsically linked to the proficiency of the personnel involved. As previously noted, any intervention that fails to cause immediate death will likely result in acute suffering that might only be captured by CORT data if the animal survives beyond this latency period. Therefore, while CORT levels provide a standardized metric of the physiological response to alternative management methods, they should be interpreted as a single facet of the animal’s experience rather than a definitive indicator of its subjective state. Future studies incorporating more immediate biomarkers, although logistically demanding in field settings, would further refine our understanding of the sensory experiences associated with different culling techniques. Unlike trapping, evidence-based approaches are rarely applied to the selection and use of shooting methods. Instead, there has been a generic transfer of methods from recreational hunting to professional wildlife management (Caudell *et al.*, 2009). Therefore, to ensure ethical shooting practices, comprehensive shooter education—including the ethical aspects of wildlife management—and rigorous testing tied to firearms certificates would likely enhance animal welfare. Also, studies are needed to evaluate animal welfare across different shooting techniques involving different types of firearms or pellet calibre. It is also crucial to stay updated on emerging technologies that allow for more accurate, quieter and effective shots.

Finally, it is important to note that since management interventions for avian invaders often include population reduction through lethal control, scenarios of social disputes and conflicts are becoming more frequent. These conflicts can complicate management efforts and lead to undesirable environmental and social outcomes (Crowley, 2021). Our findings provide a critical physiological basis to address these concerns, demonstrating that lethal shooting, while often socially contested, represents a significant refinement in terms of reducing the duration and magnitude of the systemic stress response compared to trapping methods. In such scenarios, the effective and ethical management of avian invaders needs a comprehensive communication strategy designed to assess public perception of the invasive species, to educate the public on the need for control measures to mitigate future damage and to facilitate the involvement of sensitive societal groups in management decision-making processes (Postigo *et al.*, 2019). By incorporating objective metrics, such as the endocrine profiles presented here, managers can offer transparent, evidence-based justifications for the selection of control techniques, potentially reducing social conflict and fostering greater public acceptance of refined lethal management protocols.

In conclusion, our results carry important implications for management. Since shooting minimizes physiological strain as measured by glucocorticoid response, coupled with its proven efficacy in reducing population numbers, we strongly advise practitioners to prioritize its use for controlling invasive psittacine species. In addition, we recommend raising public awareness about the varying physiological impacts and the duration of the stress response associated with different bird control methods, as a way of easing the implementation of evidence-based control methods.

## Supplementary Material

Web_Material_coag025

## Data Availability

The raw data supporting the conclusion of this article will be made available by the authors, without undue reservation. I.L.R. should be contacted if someone wants to request the data from this study.
