# Supplementary material for: Assessing animal welfare in invasive bird management: evidence for reduced stress hormones with lethal shooting
Source: Conserv Physiol. 2026 Apr 22;14(1):coag025. doi: 10.1093/conphys/coag025 (PMC13102500; doi:10.1093/conphys/coag025)
Supplement: Web_Material_coag025 [file web_material_coag025.zip › Supplementary Material_clean.docx]

**Supplementary Material**

**Figure S1.** Results of a preliminary test to validate the kit specificity to the study species. The standard curve (blue) and the monk parakeet plasma serial dilution (orange) show high parallelism and linearity. Equations for each line clearly indicate similarity of slopes.


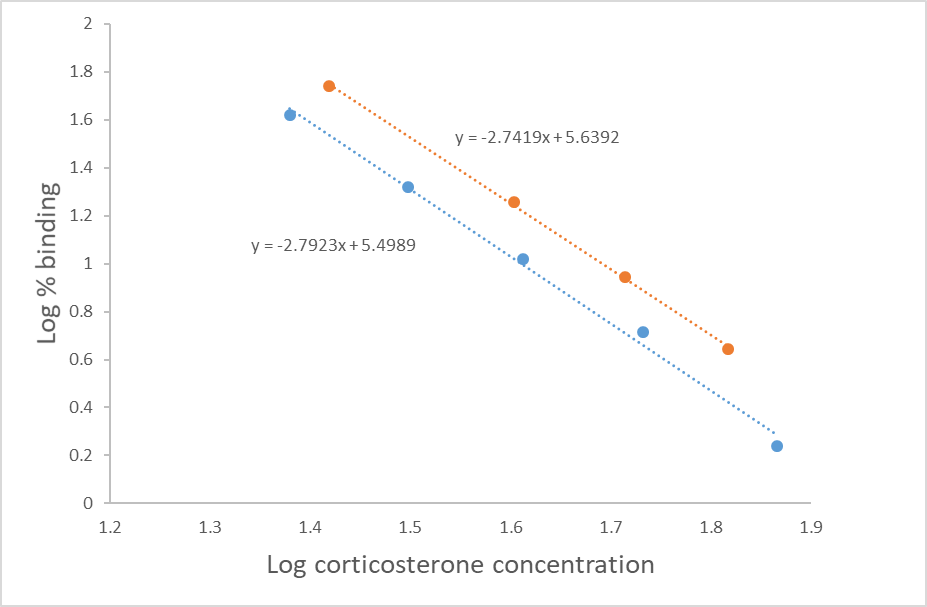


**Table S1.** a) Estimated model coefficients for the non-lineal model with Gamma error distribution assessing the effect of treatment on CORT levels in monk parakeets (*Myiopsitta monachus*). Standard errors (Std. Error), and p values for each model coefficient are provided. b) Pairwise comparisons of treatments using post-hoc Bonferroni tests.

1. **Model**

| **Coefficient** | **Estimate** | **Std. Error** | **t value** | **Pr (t)** |
| --- | --- | --- | --- | --- |
| Intercept | 3.994 | 0.590 | 6.760 | < 0.001 |
| Factor1 (live trapping) | 37.041 | 2.347 | 15. 847 | < 0.001 |
| Factor 2 (Shooting) | -1.618 | 0.702 | -2.304 | 0.025 |

1. **Pairwise comparisons**

| **Comparison** | **Ratio (exp(β))** | **IC 95%** | **P value** |
| --- | --- | --- | --- |
| Baseline-Live trapping | -37.04 | (-42.82, -31.26) | <0.001 |
| Baseline-Shooting | 1.62 | (-0.11, 3.35) | 0.074 |
| Live trapping- Shooting | 38.66 | (32.99, 44.33) | <0.001 |

**Table S2.** Estimated model coefficients for the logistic (a) and quadratic (b) models assessing the effect of time since capture on CORT levels in monk parakeets (*Myiopsitta monachus*). Standard errors (Std. Error), and significance tests (Pr(z)) for each model coefficient are provided.

| **a) Logistic model** | | | | |
| --- | --- | --- | --- | --- |
| **Coefficient** | **Estimate** | **Std. Error** | **z value** | **Pr(z)** |
| a | 43.945 | 2.641 | 16.639 | < 2.2e-16 |
| b | 0.132 | 0.022 | 5.917 | 3.27e-09 |
| x0 | 21.021 | 3.263 | 6.442 | 1.18e-11 |
| **b) Quadratic model** | | | | |
| **Coefficient** | **Estimate** | **Std. Error** | **t value** | **Pr(t)** |
| a | 1.667 | 0.346 | 4.816 | 1.03e-05 |
| b | 0.881 | 0.068 | 12.893 | < 2e-16 |
| c | -0.003 | 3.918e-04 | -8.949 | 1.20e-12 |

**Table S3.** Model comparison results evaluating the effect of sex on the CORT response of monk parakeets (*Myiopsitta monachus*) over time since capture. Eight logistic models were fitted and compared. The null model assumed no sex differences in the CORT response. Seven additional models allowed parameters of the logistic function (‘x0’, ‘a’, and ‘b’) to differ between males and females, including various combinations of these parameters. Comparisons were based on the Akaike Information Criterion corrected for small sample size (AICc). Res. Df = Residual degrees of freedom; ΔAICc = Differences in AICc relative to the best-fitting model (i.e., the model with the lowest AICc); Df = Number of estimated parameters; Weight = **relative likelihood** of each model being the best among the candidate set, given the data; Pseudo-R² = proportion of variance explained by each model. Best models are shown in bold.

| **Model** | **Res. Df** | **AICc** | **ΔAICc** | **Df** | **Weight** | **Pseudo-R^2^** |
| --- | --- | --- | --- | --- | --- | --- |
| **No sex differences** | **60** | **456.651** | **0.124** | **4** | **0.286** | **0.593** |
| Sex differences in ‘a’ | 59 | 459.923 | 3.396 | 5 | 0.053 | 0.594 |
| **Sex differences in ‘x0’** | **59** | **458.411** | **1.884** | **5** | **0.118** | **0.578** |
| **Sex differences in ‍‘b’** | **59** | **456.527** | **0.000** | **5** | **0.304** | **0.593** |
| ‍Sex differences in ‘a’ and ‘b’ | 58 | 464.010 | 7.484 | 6 | 0.007 | 0.553 |
| **‍Sex differences in ‘a’ and ‘x0’** | **58** | **456.685** | **0.158** | **6** | **0.281** | **0.624** |
| Sex differences in ‘b’ and‍ ‘x0’ | 58 | 465.245 | 8.714 | 6 | 0.004 | 0.572 |
| Sex differences in ‘a’, ‘b’ and ‘x0’ | 57 | 471.772 | 15.245 | 7 | <0.001 | 0.515 |
